# MicroRNA Expression Profiling in Porcine Liver, Jejunum and Serum upon Dietary DON Exposure Reveals Candidate Toxicity Biomarkers

**DOI:** 10.3390/ijms222112043

**Published:** 2021-11-07

**Authors:** Maia Segura-Wang, Bertrand Grenier, Suzana Ilic, Ursula Ruczizka, Maximiliane Dippel, Moritz Bünger, Matthias Hackl, Veronika Nagl

**Affiliations:** 1 BIOMIN Research Center, BIOMIN Holding GmbH, Technopark 1, 3430 Tulln, Austria; maia.segura-wang@dsm.com (M.S.-W.); bertrand.grenier@dsm.com (B.G.); suzana.ilic@dsm.com (S.I.); 2 University Clinic for Swine, Department for Farm Animals and Veterinary Public Health, University of Veterinary Medicine Vienna, Veterinaerplatz 1, 1210 Vienna, Austria; ursula.ruczizka@vetmeduni.ac.at (U.R.); maximiliane.dippel@vetmeduni.ac.at (M.D.); moritz.buenger@vetmeduni.ac.at (M.B.); 3 TAmiRNA GmbH, Leberstrasse 20, 1110 Vienna, Austria; matthias.hackl@tamirna.com

**Keywords:** deoxynivalenol, mycotoxin, microRNA, next generation sequencing, biomarker

## Abstract

Deoxynivalenol (DON), a frequent mycotoxin worldwide, impairs human and animal health. The response of microRNAs, small non-coding RNAs, to DON has been scarcely investigated, but holds remarkable potential for biomarker applications. Hence, we aimed to investigate DON-induced changes in the microRNA expression in porcine liver, jejunum and serum by combining targeted and untargeted analyses. Piglets received uncontaminated feed or feed containing 900 µg/kg and 2500 µg/kg DON for four weeks, followed by a wash-out period. In tissue, only slight changes in microRNA expression were detected, with ssc-miR-10b being downregulated in liver of DON-exposed piglets. In serum, several microRNAs were differentially expressed upon DON exposure, four of which were validated by qPCR (ssc-miR-16, ssc-miR-128, ssc-miR-451, ssc-miR-205). The serum microRNA response to DON increased over time and declined after removal of contaminated diets. Receiver operating curve analyses for individual microRNAs were significant, and a combination of the four microRNAs increased the predictive capacity for DON exposure. Predicted microRNA target genes showed enrichment of several pathways including PIK3-AKT, Wnt/β-catenin, and adherens junctions. This study gives, for the first time, a comprehensive view of the porcine microRNA response to DON, providing a basis for future research on microRNAs as biomarkers for mycotoxins.

## 1. Introduction

Mycotoxins, secondary metabolites of fungi, are frequent contaminants of food and feed that impair human and animal health. Deoxynivalenol (DON), as other trichothecene mycotoxins, is a sesquiterpenoid containing a 9, 10 double bond and a 12, 13 epoxide group [1]. DON is produced by various *Fusarium* spp. and is one of the most prevalent mycotoxins worldwide. For example, in a recent survey, 60% of feed and feed raw materials were contaminated with DON, with maximum levels exceeding 80,000 µg/kg [2]. The major mode of action of DON is binding to the 60S subunit of the ribosome and subsequent inhibition of protein synthesis [3].

Among livestock species, pigs are particularly prone to the effects of DON which is attributed to species-specific differences in phase I and II metabolism [4]. The gastrointestinal tract represents the first target of DON in pigs, where this mycotoxin impairs the gut barrier, alters the absorptive functionality or modulates the local immune response [5]. On a systemic level, DON reduces the feed intake of pigs and causes feed refusal and vomiting at high concentrations [3]. As a consequence, performance parameters, such as body weight gain, can be markedly reduced in DON exposed pigs which results in considerable economic losses [6]. Despite its relevance for animal production, diagnosis of DON-induced disorders in pigs is still challenging. This is mainly due to the absence of appropriate biomarkers that are applicable in the field [4]. Further elucidation of the molecular mode of action of DON might facilitate the identification of new molecules that respond to this mycotoxin and allow non- or minimal-invasive sampling of animals.

In this respect, microRNAs represent an interesting target. These small (approximately 22 nucleotides long), non-coding RNAs act as negative regulators of gene expression [7]. Since the discovery of the first microRNAs in the 1990s, the scientific interest in the role of microRNAs has constantly increased which stems from certain toxicological features inherent to microRNAs [8]. First, microRNAs are actively or passively released from tissues into extracellular biofluids, such as blood, urine or saliva. Hence, pathological processes in tissues induced by environmental changes or exposure to toxicants can be detected via measurement of circulating microRNAs in biofluids, which are easily accessible for sampling [9,10]. In addition, the specificity and stability of microRNAs represent advantages compared to the measurement of other biomolecules, e.g., proteins or mRNAs [8]. While in human medicine the research on microRNAs spans from investigating the role of microRNAs in the pathogenesis of multiple diseases, over their utilization as biomarkers to their potential application as therapeutics, research on microRNAs in livestock species is comparably limited [11].

In recent years, first reports on the involvement of microRNAs in the toxicodynamics of mycotoxins became available. Although the role of microRNAs in mycotoxin toxicity was highlighted, reports primarily address the mycotoxin aflatoxin B1 or ochratoxin A and employ in vitro settings [12]. In contrast, studies investigating the relevance of microRNAs in DON toxicity are scarce (Table S1), even more so in pigs. Xie et al. [13] and Hou et al. [14] examined the effects of DON on selected microRNAs in porcine intestinal epithelial cells (IPEC-J2) in vitro and explored potential strategies to counteract DON-induced intestinal damage. Out of a total of 13 microRNAs analyzed in these two studies, effects of DON were observed for five microRNAs in IPEC-J2 cells, namely miR-92a, miR-221, miR-148a, miR-185 and miR-222 [13,14]. To the best of our knowledge, there is only one literature report available investigating the influence of DON on porcine microRNA expression in vivo. Brzuzan et al. [15] measured selected microRNAs (miR-9, miR-15a, miR-21, miR-34a, miR-122, miR-125b, miR-192) in different sections of the gastrointestinal tract in gilts, revealing upregulation of miR-21 in the ascending colon after seven days of DON exposure and downregulation of miR-15a in liver after 21 days of DON exposure. So far, experiments employing next generation sequencing (NGS) technologies to unveil the impact of DON on the global microRNA expression in pigs are lacking. It has been shown that pigs and humans present similar processes of mycotoxin absorption, distribution, metabolism and excretion, which are essential characteristics to understand, assess and extrapolate the toxicological effects of mycotoxins [16]. Given the importance of DON for pig production, the high sensitivity of pigs towards mycotoxins, as well as the comparably high suitability of pigs as animal models for assessing the toxicological risk of mycotoxins in humans [16], studies in this species are of relevance.

Thus, the objective of our study was to explore the effects of dietary DON exposure on the genome-wide microRNA expression in liver, jejunum and serum of weaned piglets. We hypothesized that DON-induced alterations of intestinal or hepatic microRNA expression are reflected in the pool of circulating microRNAs in serum. Surprisingly, the impact of DON on tissue microRNA expression was less pronounced compared to serum. Combining untargeted and targeted approaches for analysis of circulating microRNAs, we confirmed four microRNAs (ssc-miR-16, ssc-miR-128, ssc-miR-451, ssc-miR-205) being significantly upregulated upon DON treatment in serum. The present research provides, for the first time, comprehensive information on the microRNA response to dietary DON in pigs, thereby stimulating further research on the involvement of microRNAs in DON toxicity and their potential application for DON exposure assessment.

## 2. Results

### 2.1. DON Induces Only Slight Changes in microRNA Expression in Jejunum and Liver

**Figure 1:**
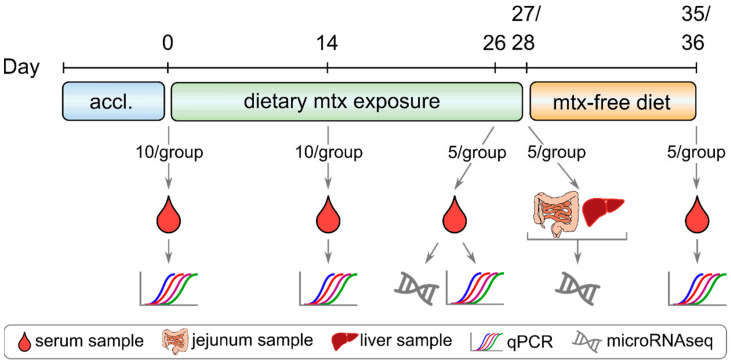
Sample collection scheme. Sample types, days of sampling and analyses performed are shown (accl.: acclimatization phase; mtx: mycotoxin).

**Figure 2:**
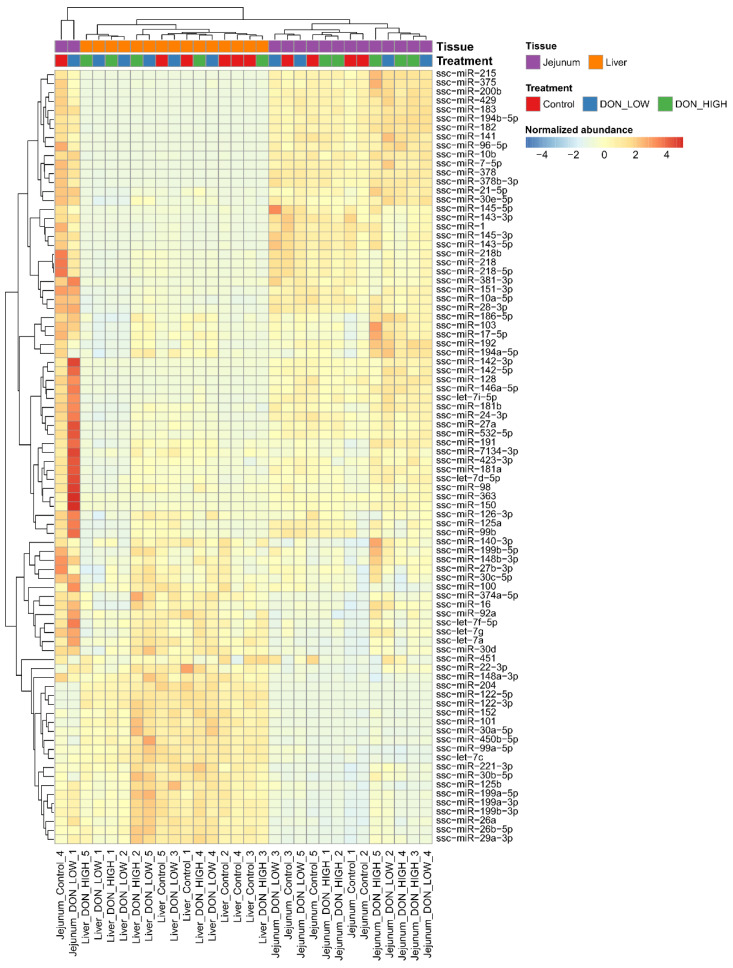
MicroRNA expression profiles of Control and DON exposed pigs in liver and jejunum. Heatmap of microRNA expression in all samples from the Control and DON exposed pigs.

After four weeks of exposure to different treatment diets (Control group: uncontaminated feed, DON_LOW: 900 µg DON/kg feed, DON_HIGH: 2500 µg DON/kg feed), jejunum and liver samples from piglets were subjected to small RNA sequencing (the complete sampling scheme is shown in Figure 1). In total, between 6.5 and 13 million reads were generated for each tissue sample. On average, after quality filtering and size selection, 80% of the mapped reads were identified as microRNA (Table S2), and 348 different microRNAs detected per sample. The microRNA abundance profiles were clearly different for each tissue, with jejunum samples clustering together and separate from liver samples, but no clear separation by the different treatments was observed (Figure 2). Principal component analysis (PCA) revealed similar results, with no clear clustering of samples according to treatment, neither for jejunum nor for liver samples (Figure S1b,c).

Given that the microRNA expression was shown to be different in jejunum and liver, samples were analyzed separately according to their tissue of origin. Differential expression analysis between the DON_LOW or DON_HIGH and the Control samples revealed very small differences in microRNA expression in jejunum and liver induced by the DON treatment. For jejunum samples, no significantly differentially abundant microRNA was found, based on a False Discovery Rate (FDR) < 10%, when comparing DON_HIGH treated samples to the Control samples nor between the DON_LOW samples and the Controls. For liver samples, one microRNA, ssc-miR-10b, was shown to be significantly downregulated (FDR < 10%) in the DON_HIGH treatment group (Figure S2). No other microRNAs were found to be significantly different in the liver samples when correcting for multiple testing.

### 2.2. DON Induces Significant Differential microRNA Expression in the Serum

**Figure 3:**
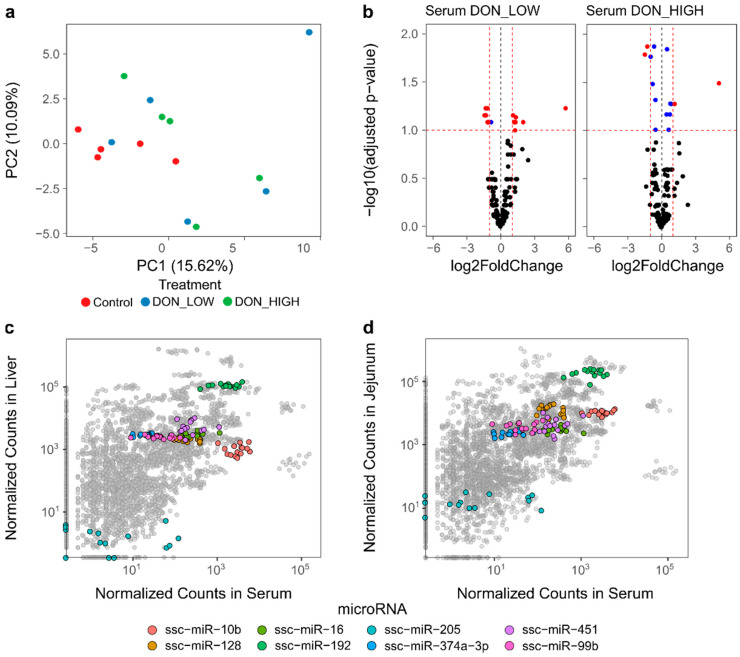
MicroRNA abundance in serum samples based on sequencing data. (**a**) Principal component analysis based on microRNA abundance in the serum samples; (**b**) volcano plots of differentially expressed genes showing the fold changes and FDR (adjusted *p*-values) of microRNAs in the DON_LOW and DON_HIGH samples compared to Controls. Red dots: microRNAs with FDR < 10% and > 2-fold change; blue dots: microRNAs with FDR <10%; (**c**) normalized counts in liver and serum for all microRNAs in all analyzed samples; (**d**) normalized counts in jejunum and serum for all microRNAs in all samples. The eight selected microRNAs found in liver as well as in jejunum are highlighted. Each dot represents the abundance of a microRNA in a specific sample.

To assess whether the microRNA expression response observed in liver could also be detected in the serum (as a minimal-invasive marker for DON toxicity), or if other patterns of expression could be identified, sequencing data from serum samples of the same animals were generated. For these serum samples, the total sequencing read counts per sample after adapter trimming and size filtering ranged from 3 to 20 million reads, with an average of 3.5% of the mapped reads classified as microRNAs and an average of 226 different microRNAs detected per sample. Accumulation curves of the relation between the microRNA read count (i.e., reads mapping to microRNAs) and the number of microRNAs identified in the samples showed that there was a comparable and comprehensive recovery of microRNAs, with very few additional microRNAs detected with increasing sequencing depth. Similar to the results for the tissues, no clear clustering of samples was observed (Figure 3a), which indicated that the information of the global microRNA abundance was not sufficient to cluster the samples by their treatment groups.

To identify individual microRNAs with significant differences in abundance between the treatment groups, differential abundance analysis was performed. In total, 15 microRNAs (DON_LOW vs Control) and 16 microRNAs (DON_HIGH vs. Control) were significantly altered after DON exposure in the serum (Figure 3b and Table S3). Among all the differentially abundant microRNAs identified, eight were significantly altered in both DON_LOW and DON_HIGH. Out of these, four microRNAs were downregulated upon DON treatment (ssc-miR-10b, ssc-miR-99b, ssc-miR-192, ssc-miR-374a-3p), and four were upregulated in DON exposed pigs (ssc-miR-16, ssc-miR-128, ssc-miR-205, ssc-miR-451) (Table 1 and Table S4). For the four upregulated microRNAs, strong increases in their abundance, of up to 54 times (for ssc-miR-128), were observed even in the DON_LOW group. Ratios were generated between all these eight microRNAs, and receiver operating characteristic (ROC) curve analysis was performed to examine whether microRNA ratios could be used as classifiers of non-exposed (Control) and treated (DON_LOW, DON_HIGH) pigs. Out of 16 ratios evaluated, 13 showed area under the curve (AUC) values higher than 0.9 (*p* < 0.01) implying that they represent good classifiers for DON exposure, with the best ratio being ssc-miR-128/ssc-miR-374a-3p (Figure S3). Even when testing these ratios as classifiers of non-exposed (Control) and moderately exposed (DON_LOW) animals, significant effects were found (data not shown).

The relation between the abundance of microRNAs in the serum compared to the abundance in the liver and jejunum was investigated to evaluate if differentially expressed microRNAs had a similar trend between the different sample sources. No correlation between the abundance of microRNA in serum and in liver was observed (Pearson *r* = 0.01; *p* > 0.05) (Figure 3c), nor between serum and jejunum (Pearson *r* = 0.03; *p* > 0.05) (Figure 3d), confirming the unique microRNA expression profiles in different tissues. Larger variation in the expression values were seen in the serum (broader spread in the *x*-axis), and less variation in the liver or jejunum samples was observed. There were no significant differences in abundance found in these microRNAs in the liver nor in the jejunum. MicroRNA ssc-miR-10b, which showed significant differences in the liver of the DON_HIGH group, was also significantly decreased in the serum of DON exposed pigs, both in the DON_LOW and DON_HIGH group. In serum, this microRNA was one of the most abundant microRNAs (Figure 3c,d). In general, no relation between microRNA abundance and differential expression was observed, indicating that not only highly expressed microRNAs were detected as differentially expressed.

### 2.3. Upregulation of microRNA Expression in the Serum Was Validated by qPCR

**Figure 4:**
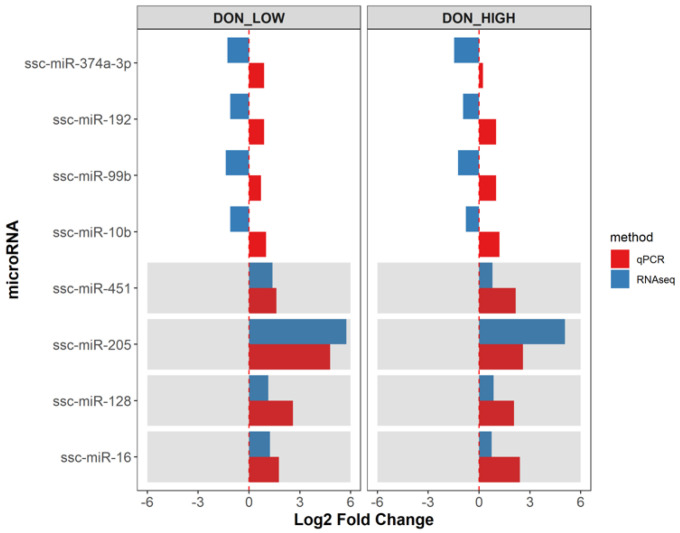
Comparison of microRNA expression in the serum based on qPCR and sequencing data. Log2 fold changes of in the DON_LOW (left) and DON_HIGH (right) compared to the Control group assessed via small RNA sequencing (blue) and qPCR (red).

The eight microRNAs that were altered in both treatments, DON_LOW and DON_HIGH, based on sequencing data of serum samples at day 26 were selected for further qPCR validation (Figure 1). Using data from the same animals for which sequencing data was available, the changes in expression upon DON treatment were estimated based on the qPCR results. The log2 fold changes of microRNAs in the DON_LOW and DON_HIGH groups are shown in Figure 4. The upregulation of ssc-miR-16, ssc-miR-128, ssc-miR-205, and miR-451 was confirmed via qPCR, with similar trends observed based on sequencing and qPCR. In line with these results, there was a highly significant correlation between sequencing data and qPCR output (normalized reads vs. normalized Ct) for the four upregulated microRNAs (all with *p* < 0.05; Spearman correlations) (Figure S4). In contrast, for the four miRNAs that were downregulated based on the sequencing data (ssc-miR-10b, ssc-miR-99b, ssc-miR-192, ssc-miR-374a-3p), the qPCR results showed less pronounced differences in expression. Furthermore, the downregulated microRNAs did not show a significant correlation between the abundance measured by the two methods (Figure S4). Consequently, the microRNAs that were initially identified as downregulated based on the sequencing data could not be validated by qPCR.

### 2.4. DON Effects on microRNA Expression Increase with Longer Exposure Times

**Figure 5:**
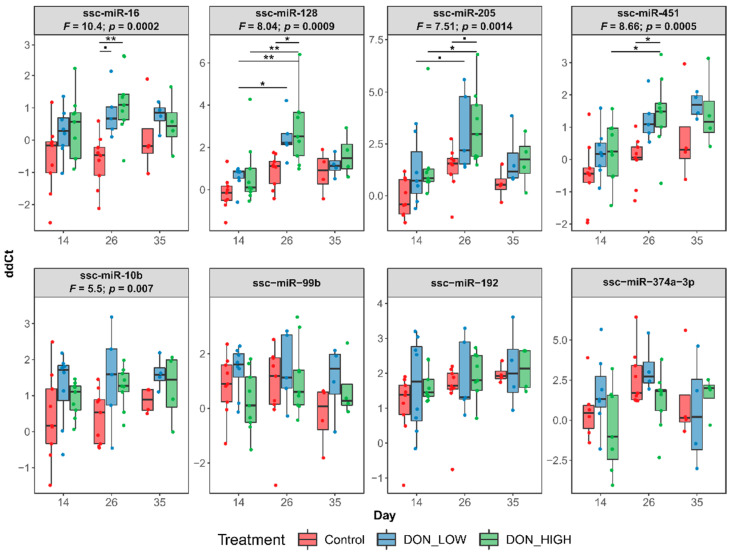
MicroRNA expression in the serum based on qPCR. Comparison of microRNA expression in different treatment groups, normalized by the baseline expression at d0. Significant values for the treatment effect are shown above each plot based on 2-way ANOVA followed by Tukey’s multiple comparison test. Significant differences are marked as follows: ■ *p* < 0.1; * *p* < 0.01; ** *p* < 0.001.

The effect of DON exposure overtime was evaluated by measuring microRNA expression in the serum at additional time points by qPCR, comprising days 0, 14, and 35, in addition to the data of day 26 presented above. Absorbance at 414 nm measured in all samples before qPCR analysis showed six potentially hemolytic samples that were removed from downstream analyses (Figure S5). At day 0, before samples had received a DON contaminated diet, some variability in the microRNA abundance was observed (Figure S6). Therefore, for comparing microRNA abundance overtime, the Ct-values were normalized against the starting abundance at day 0. Overall, for the upregulated microRNAs, there was a significant difference between DON treated samples compared to the Controls (for each upregulated microRNAs *p* < 0.01; two-way ANOVAs), with increased abundance in the treated samples (Figure 5). Already on day 14, an upregulation of ssc-miR-16, ssc-miR-128, ssc-miR-205, and ssc-miR-451 was noticeable in DON-exposed piglets (Figure 5). On day 26, the microRNAs ssc-miR-16, ssc-miR-128, and ssc-miR-451 were significantly higher in DON_HIGH treated samples compared to Controls (between *p* < 0.01 and *p* < 0.001; Tukey’s multiple comparison tests), and a similar trend was observed for ssc-miR-205 (*p* < 0.1). Although not significant, also an increase in microRNA abundance was observed in the in DON_LOW treated samples. Furthermore, the upregulated microRNAs showed an increase in abundance compared to the previous time point, with a significantly higher abundance of ssc-miR128, ssc-miR-205 and ssc-miR-451 (*p* < 0.01) and a similar trend for ssc-miR-16 at day 26 than at day 14, pointing to a stronger effect of DON in the abundance of these microRNA with longer periods of contaminated feed intake. On day 35 (i.e., after toxin removal), the microRNA response in previously DON-exposed piglets was numerically reduced compared to the Control group. Therefore, the effect of DON on these microRNAs seems to be reversible, with reduced abundance upon removal of the toxin.

### 2.5. Four microRNAs in Serum Effectively Classify DON Exposed Pigs

**Figure 6:**
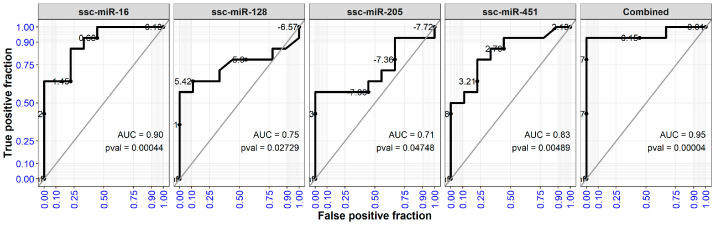
ROC analysis based on microRNA expression measured by qPCR for serum samples at day 26. For this analysis, DON_LOW and DON_HIGH samples were pooled together as the treated group and compared to the Control. The Combined ROC curve shows the analysis based on the logistic regression model using a combination of these four microRNAs.

The four upregulated microRNAs were evaluated in terms of their ability to differentiate between Controls and DON exposed pigs (DON_LOW and DON_HIGH together). ROC curve analysis on the microRNA abundances in the serum at day 26 based on qPCR measures showed that ssc-miR-16 and ssc-miR-451 exhibited significantly high AUCs of 0.90 and 0.83, respectively (*p* = 0.0004 and *p* = 0.005). Similarly, ssc-miR-128 and ssc-miR-205 also showed elevated AUCs (*p* < 0.05) (Figure 6). These results indicated that each of these individual microRNAs could be used independently to identify DON exposed pigs. Additionally, multiple logistic regression using the combination of the four serum microRNA expressions followed by ROC curve analysis on the regression model showed a higher predictive value (AUC = 0.95, *p* = 0.00004) compared to any of the single serum microRNAs, which indicated that combining the information from these microRNAs was a better way to distinguish DON effects.

### 2.6. MicroRNA Expression Does Not Correlate with Animal Performance

The effects of DON on performance parameters of piglets included in this study were reported in detail by Bünger et al. [17]. In short, dietary DON exposure did not result in statistical differences in the body weight of the piglets, and clinical signs of DON-mycotoxicosis, such as feed refusal, were absent. The body weight of the piglets and the microRNA abundance showed no correlation based on the sequencing data or on the qPCR data (Figure S7), indicating that the microRNA abundance changes observed in the current study were not influenced by the weight of the animals.

### 2.7. Target Genes of Upregulated microRNAs Are Enriched for Critical Signaling Pathways

To better understand the biological functions of the four upregulated microRNAs, the potential target genes of these microRNAs were predicted. In total, 662 target genes from all microRNAs were predicted by both the miRTarBase database and the microT-CDS tool. The majority of these genes, 96%, were uniquely targeted by a single microRNA. These predicted genes were mostly contributed by the ssc-miR-16, with 435 targets, followed by ssc-miR-128 with 176 targets. The highest overlap of target genes was between ssc-miR-16 and ssc-miR-128, with 13 genes. There was a single gene target of three microRNAs, coding for the transcription factor AFF4 (Figure S8).

The microRNA target genes were mapped in Kyoto Encyclopedia of Genes and Genome (KEGG) pathways and an enrichment analysis on the gene union was performed. In total, 87 different KEGG pathways were significantly enriched (FDR < 0.05), related to a wide range of functions (Table S5). Given that the target gene predictions were based on human data, many enriched pathways were related to cancer. Nevertheless, many other functions were additionally identified, including several signaling pathways: PI3K-AKT (phosphatidylinositol 3-kinase/AKT pathway), FoxO (forkhead box O pathway), mTOR (mammalian/mechanistic target of rapamycin pathway), Wnt (Wnt/β-catenin pathway) and MAPK (mitogen-activated protein kinases pathway), the adherens junction pathway, and the fatty acid biosynthesis pathway. Network analysis of the enriched gene-sets showed that multiple identified pathways were highly interconnected (Figure S9). Furthermore, the biological functions of the target genes classified based on Gene Ontology (GO) terms and grouped in high-level GO terms showed that the largest functional group was “Regulation of biological quality”, with 199 genes, and interestingly the second largest was “Response to stress”, with 198 genes (Table S6).

## 3. Discussion

DON is considered as one of the most relevant mycotoxins contaminating food and feed, thereby affecting human and animal health. Its physiological effects have been assessed through different methods, but its toxicity at the molecular level and mechanisms of action are not fully understood. In this regard, microRNAs have become promising molecular markers for the diagnosis and monitoring of specific diseases, especially through the use of next-generation sequencing technologies, due to their stability and tissue specificity. Additionally, microRNAs have highly conserved sequences and share target genes even between distant species, indicating that similar pathways and biological processes may be regulated by the same microRNAs [9,18]. Nevertheless, there is limited information about the effects of DON on microRNA expression in vivo. Consequently, in this study, we investigated the impact of dietary DON exposure on the microRNA expression profiles in porcine jejunum, liver, and serum. Albeit conducted under experimental settings, the DON concentrations in feed and the exposure duration over several weeks were chosen to mimic practical conditions. More specifically, the DON_LOW group was administered feed containing 900 µg/kg DON, which reflects the recommendation of the European Commission for maximum DON levels in pig feed [19]. To study potential dose-dependent effects of DON on microRNA expression in pigs, feed containing 2500 µg/kg DON was used for the DON_HIGH group. Although this concentration exceeds the EC recommendations, it still represents a contamination level to be found under field conditions [20], where maximum levels exceeding 32,000 µg/kg can be encountered in finished feed samples [2].

To the best of our knowledge, only one in vivo study was published about the effects of DON on microRNA expression in piglets, which suggested only small DON-induced differences in a limited set of seven microRNAs [15]. These results are consistent with the rather minor changes in the microRNA expression profiles in the jejunum and liver observed in the present study. Similarly, based on PCA, no obvious clustering of samples according to treatment was observed, indicating that the information of the microRNA expression in liver and jejunum was not enough to group the samples into their treatment categories. Even when investigating the effects of DON on the global microRNA expression levels, only one microRNA, ssc-miR-10, showed significant differences in the liver. This was unexpected given that the intestine and liver have been reported as one of the major target organs damaged by exposure to DON [5,21]. For example, in piglets exposed to feed contaminated with 3000 µg/kg DON for five weeks, significantly increased lesion scores in jejunum and liver were determined [22]. Even though few DON-induced differences were observed in the respective tissues based on microRNA expression in the present study, we showed microRNA expression differences depending on the tissue of origin, as expected from the diverse regulatory processes in the different tissues [23].

In serum, more microRNAs were significantly affected by DON treatment compared to jejunum or liver. Surprisingly, none of the eight selected microRNAs in the serum showed correlations with the jejunum or liver. This indicates that changes in circulating microRNAs did not stem from microRNAs released from liver or jejunum, but potentially from tissues not investigated in this study (e.g., spleen) or blood cells. For example, it has been reported that several immune cells types including monocytes, macrophages, and T- and B-lymphocytes are targets of DON [1,24], and these effects might then drive the observed systemic changes. However, looking at the overall correlation of microRNAs between serum and liver or jejunum in the present study, our dataset revealed correlation coefficients in the same low range as the ones reported by Cui & Cui [25], suggesting that there is in general a similar trend between the tissue and the serum expression, but this relation is not strong for the eight specific microRNAs selected. Brzuzan et al. [15] compared the expression of seven microRNAs in porcine liver, duodenum, and jejunum after DON exposure and observed expression profiles specific for each tissue. However, no comprehensive data of microRNA expression at a global scale is available for pig liver, jejunum or serum after DON exposure. Our findings are among the first to provide these tissue comparisons, highlighting the importance of future studies to better understand the relation between circulating microRNAs and specific conditions in tissues. Nevertheless, further studies are needed to identify the exact tissues or cell types of origin of these microRNAs.

The microRNA expression differences between serum samples from DON exposed pigs and Controls were confirmed by qPCR analysis, albeit only for microRNAs being upregulated upon DON exposure. It was interesting that, for the downregulated microRNAs identified based on the sequencing data, the qPCR analysis provided opposite results. Tao et al. [26] evaluated microRNA expression by qPCR and microarray in porcine serum and found similar trends with both methods, although lower fold-changes were seen in the qPCR data. Variation of the results could be likely due to the different sensitivities of both methods, or to different normalization approaches in the NGS-based and qPCR-based analyses, but further comparisons are needed using similar methods and larger sample sizes to better understand the discrepancies. Nonetheless, the qPCR expression values for ssc-miR-16, ssc-miR-128, ssc-miR-451, and ssc-miR-205 proved to be effective in distinguishing between DON exposed and non-exposed pigs independent of the DON concentration, both as single measures and in combination, supporting their use as candidate biomarkers for assessment of DON effects. Additionally, the lack of significant correlation with animal performance indicated that the microRNA alterations were not influenced by the body weight of piglets.

In addition to the identification and validation of four upregulated microRNAs in pig serum, we showed a significant effect of the duration of exposure to DON on microRNA expression indicating that the effect of DON could be relatively fast and temporal. Based on human cell culture data, DON effects depend on the dose and length of exposure [19], derived from differences in the intensity and duration of kinase signaling affecting gene expression [24]. Prolonged DON feeding in mice causes higher levels of serum IgA, indicating that, with longer exposure times, there is a more pronounced effect [27]. In accordance, the microRNA response of DON exposed pigs increased over time in the present trial. However, it should be noted that prolonged monotonic mycotoxin exposure does not necessarily result in more distinct alterations of serum biochemical parameters as compensatory mechanisms can take place [28]. Furthermore, our data suggest that the microRNA expression is transiently modified while the animals are under DON stress, but values tend to return to a normal state after toxin removal. Given the rapid absorption and elimination of DON in pigs [1], those results are not surprising. However, to the best of our knowledge, dynamic changes in microRNA expression (or in levels of other potential serum biomarkers) have not been previously studied after removal of DON contaminated diets in pigs which hampers comparisons to literature. The rapid normalization of microRNA expression after removal of DON is in line with a fast transcriptional activation and stabilization of mRNAs in the DON proinflammatory response in human leucocytes [3].

MicroRNAs are suggested to play important roles in the toxicodynamics of mycotoxins, including DON, through the regulation of the expression of target genes and enzymes involved in physiological responses to diseases and toxic agents [12]. The broad impact of ssc-miR-16 was evident based on its high number of target genes. This microRNA has been observed ubiquitously in almost all porcine tissues, suggesting its important role in constitutive processes [23,29]. Consequently, its upregulation can have extensive effects on the organism, including its association with stress response in pigs [11]. Upregulation of ssc-miR-205 expression has been also reported based on microarray experiments in pig serum (among 10 core microRNAs) upon weaning stress [26]. These authors found consistent results for ssc-miR-205 also in jejunum of the stressed pigs. Notably, toxic effects of DON comprise induction of oxidative stress and reactive oxygen species [30,31,32]. In the same line, high levels of mirR-128, as we observed in the present study in DON exposed pigs, have also been associated with higher oxidative stress [33].

Given the lack of experiments investigating the microRNA expression profile upon DON treatment in pigs, we further explored the potential biological impacts of the upregulation of these microRNAs by examining enriched functional pathways. Several studies have previously shown that DON impacts critical signaling pathways, and thereby has been associated with several molecular effects, including induction of cytotoxicity, cell signaling and differentiation in multiple organs in humans and animals [34] and autophagy [35,36]. Furthermore, it has been postulated that DON can upregulate a large number of microRNAs, leading to the modulation of gene expression [3,12]. The enrichment of multiple signaling pathways among the target genes of the four upregulated microRNAs observed in the present study is supported by the molecular impact of DON reported in other experimental set ups. For example, in vitro studies in porcine intestinal epithelial cells (IPEC-J2) have demonstrated that DON-induced apoptosis and autophagy involves the inhibition of the PI3K-AKT and mTOR signaling pathways [35,37], known as negative regulators of autophagy. More specifically, Gu et al. [35] confirmed that, in DON treated IPEC-J2 cells, the significant downregulation of AKT and mTOR proteins was associated with cell damage and apoptosis. Our results additionally support this finding, showing molecular evidence that DON induces an upregulation of microRNA ssc-miR-16 in pig serum, which targets genes coding for proteins in the PI3K-AKT pathway including AKT. Interestingly, analogous evidence can be derived from Yin et al. [38] who reported that lead-induced apoptosis in chickens was driven by an increase in expression of miR-16, which in turn modulated the expression of genes involved in apoptosis and oxidative stress. DON-induced autophagy in IPEC-J2 cells has been also in part linked to the repression of mTOR through a phosphorylation cascade involving the AMP-activated protein kinase (AMPK) pathway [31], with several genes targeted by miR-128. Adding to the complexity of effects, there is also data from mouse spleen indicating that DON significantly modulates the phosphorylation of proteins of the PI3K-AKT pathway, triggering a cascade of events that leads to downstream immune cell activation and apoptosis that are associated with ribotoxic stress response [39]. Moreover, experimental evidence for the suppression of the Wnt signaling pathway upon DON exposure, also one of the significantly enriched pathways among the target genes, exists based on in vivo and in vitro porcine intestinal stem cells studies. By using Western Blot on a limited set of proteins, Li et al. [40] found significant suppression of the Wnt pathway upon DON exposure. In addition, Tang et al. [41] determined that DON-induced inhibition of cell proliferation involved the Wnt signaling pathway. These reports, together with our results, indicate that the suppression of the Wnt and the PI3K-AKT pathways, might be mediated through the upregulation of the confirmed microRNAs in the molecular mechanisms underlying DON cytotoxicity.

On the other hand, it has been shown that miR-205 is involved in the indirect regulation of the adherens junction protein E-cadherin in human colon and kidney as well as in porcine placenta [42,43,44]. We also observed among the target genes an enrichment of the adherens junction pathway, involved in cell–cell adhesion mainly through the action of nectins and cadherins [45]. In particular, DON is known to affect the intestinal barrier by altering the levels of tight junctions and adherens junction proteins, including cadherin. Several authors have consistently reported a downregulation or reduction of cadherins upon DON treatment on intestinal tissues in pigs [46,47,48,49], human cells [50,51], and in mice testis [52], leading to an impairment of the barrier functions. Additionally, miR-205-5p (which has the same sequence as the ssc-miR-205 upregulated in this study) is associated with the suppression of the Wnt/β-catenin pathway through the inhibition of β-catenin [53,54]. miR-205-5p suppressed the phosphorylation of p38 protein kinase resulting in the deactivation of both the MAPK and Wnt/β-catenin pathways in in vitro experiments [53], in line with the enrichment of these pathways for the microRNAs upregulated by DON, and more importantly, providing evidence of the involvement of ssc-miR-205 in DON-induced suppression of these pathways. Further studies are needed to associate the expression levels of target genes in relation to the microRNA expression driven by DON exposure. It should be kept in mind, however, as has been pointed out in other studies, that DON can induce very complex effects, as shown by the heavy interconnectivity of the genes targeted by the four microRNAs, involving the interrelation of multiple pathways with a broad impact at the molecular and physiological levels.

In conclusion, in this study, four microRNAs were identified as candidates for the detection of DON toxicity in porcine serum. The expression of these microRNAs, either combined or as single measures, was effectively used to differentiate between DON exposed pigs and Controls. Therefore, the microRNAs described here represent potential biomarkers for dietary DON exposure. Even though the general impact of DON on animal health is broadly accepted, there is a lack of toxicity markers that demonstrate its negative effects in a reproducible manner. This is especially true for experiments using realistic DON contamination levels in feed, as employed in the current study, and minimal- or non-invasive sampling. Our results show the potential of using microRNAs as effective biomarkers of DON effects through minimal-invasive sampling.

Based on the predicted target genes of affected microRNAs, we substantiate previous experimental data about the involvement of the PIK3-AKT, Wnt/β-catenin, and adherens junction pathways in DON-induced apoptosis and autophagy. Overall, these findings represent new potential targets to improve the understanding of the cellular responses associated with DON toxicity and to develop new counteracting strategies against DON effects. Future studies are required to confirm the reproducibility of our findings under various experimental conditions, evaluating potential influencing factors such as breed, age and sex of the animals as well as DON dose, exposure duration or co-contamination of feed with other (myco)toxins.

## 4. Materials and Methods

### 4.1. Animal Experiment

#### 4.1.1. Animals and Study Design

All procedures for animal handling, care and treatment of pigs have been approved by the institutional ethics committee of the Vetmeduni Vienna and the national authority according to paragraph 26 of Law for Animal Experiments, Tierversuchsgesetz 2012-TVG 2012 (BMBWF-68.205/0058-V/3b/2018). The experiment was carried out at facilities of the University Clinic for Swine, Vetmeduni Vienna. Weaned, four-week-old, female crossbred piglets (sow: Large White, boar: Pietrain) were obtained from a University-owned pig farm in Lower Austria. At arrival, piglets were allocated to different groups (*n* = 10) considering a balanced average body weight (bw) among groups (7.40 kg). Piglets were housed in pens (1 pen/group), with an area of 1.25 m^2^ per pig, on straw bedding and had free access to water and feed during the whole trial period.

After an acclimatization period of 8 days in which all piglets received uncontaminated basal feed, animals were exposed to different treatment diets for 28 days. Piglets received either uncontaminated basal feed (Control) or feed containing 900 µg/kg DON [DON_LOW, reflecting the recommendation of the European Commission for maximum DON levels in pig feed [55]] and 2500 µg/kg DON (DON_HIGH). For artificial contamination of diets, culture material of *Fusarium graminearum* was used (BiMM—Bioactive Microbial Metabolites Group, Universitäts- und Forschungszentrum, Tulln, Austria). Details regarding the mixing procedure, verification of DON levels as well as absence of other relevant mycotoxins in the diets can be retrieved from Saenz et al. [56]. At the end of the mycotoxin exposure period, necropsy of half of the piglets (*n* = 5/group) was performed on two consecutive days (d27 and d28). Independent of the treatment group, all remaining piglets (*n* = 5/group) received uncontaminated basal diet for 7 days. After this phase-out period, necropsy of remaining pigs was performed on two consecutive days (d35 and d36).

#### 4.1.2. Clinical Examination, Necropsy and Sample Collection

During the whole trial period, the general condition of the piglets as well as the occurrence of diarrhea and vomiting was assessed daily, while weighing of piglets was performed weekly. Blood samples were collected from individual piglets on d0 (prior to mycotoxin exposure), d14, d21, d26 and d35/36. After centrifugation (3756 rcf, 10 min, 20 °C), serum samples were stored at −80 °C until further analysis. Piglets were anaesthetized by intramuscular injection of Ketaminhydrochlorid (Narketan^®^, 10 mg/kg bw, Vétoquinol, Vienna, Austria) and Azaperon (Stresnil^®^, 1.3 mg/kg bw, Elanco GmbH, Cuxhaven, Germany) followed by euthanasia via intracardial injection of T61^®^ (tetracaine hydrochloride, mebezonium iodide and embutramide, 1 mL/10 kg bw, Intervet, Austria). For collection of tissue samples, the jejunum was fully unrolled. Sections of approximately 5 cm length were dissected from the middle part, rinsed with saline water and processed to pieces of approximately 0.5 × 0.5 cm² on sterile petri dishes on ice. Subsequently, samples were placed into 1 mL RNA Later (Ambion Inc., USA), stored overnight at 4 °C and transferred to −80 °C until further analysis. Liver samples were collected from the left lateral lobe and processed as described as for jejunum samples. A diagram of the sampling scheme and sample types is shown in Figure 1.

### 4.2. MicroRNA Analysis in Tissues

#### 4.2.1. MicroRNA Extraction

Jejunum and liver samples from the Control, DON_LOW and DON_HIGH groups collected on day 27/28 were subjected to small RNA sequencing (*n* = 5 per group/tissue). To this end, approximately 20–25 mg of tissue were disrupted via a bead-beating step, and the total RNA, including RNA from approximately 18 nucleotides upwards, was extracted and purified with the miRNeasy Mini Kit (Cat# 217004, Qiagen, Hilden, Germany) according to the manufacturer’s recommendation. The concentration of isolated RNAs was estimated on a NanoDrop 2000 spectrophotometer (Thermo Fisher Scientific, Vienna, Austria), and RNA quality (RIN) with an Agilent TapeStation 4200 (Agilent Technologies, Vienna, Austria).

#### 4.2.2. MicroRNA Sequencing

In total, 1 μg of total RNA was used for small RNA library preparation with the NEBNext Multiplex Small RNA Library preparation set for Illumina (New England Biolabs Inc., Ipswich, MA, USA) following the instructions of the manufacturer, selecting for insert sizes between 18 and 50 nucleotides, and using 15 PCR cycles as described by Grenier et al. [57]. The multiplexed libraries were sequenced on an Illumina HiSeq 2500 with 50 bp single-end reads.

### 4.3. MicroRNA Analysis in Serum

#### 4.3.1. MicroRNA Extraction

Serum samples from the DON_LOW and DON_HIGH groups collected on day 26 were subjected to small RNA sequencing (*n* = 5 per group, same individuals as for sequencing of jejunum/liver samples). For this, total RNA was extracted using the miRNeasy Mini Kit (Cat# 217004, Qiagen, Germany) following the instructions of the manufacturer. In short, samples were thawed at RT and centrifuged at 12,000× *g* for 5 min to remove any cellular debris. For each sample, 200 µL of serum were homogenized with 1000 µL Qiazol. To monitor RNA extraction efficiency, a synthetic RNA oligonucleotide mix from the miRCURY Spike-In kit (Cat# 339390, Qiagen, Germany) was added to each sample at equimolar amounts prior to RNA extraction. Total RNA was eluted in 30 µL nuclease free water and stored at −80 °C until further analysis.

#### 4.3.2. MicroRNA Sequencing

Total RNA (2 µL) was used for small RNA library preparation using the CleanTag Small RNA Library Preparation Kit (Cat# L-3206-24, TriLink Biotechnologies, San Diego, CA, USA). Adapter-ligated libraries were amplified for 23 cycles and sequenced on an Illumina HiSeq 2500 with 50bp single-end reads.

#### 4.3.3. Validation of microRNA Expression in Serum by qPCR

Eight microRNAs were selected based on the sequencing results (ssc-miR-10b, ssc-miR-16, ssc-miR-99b, ssc-miR-128, ssc-miR-192, ssc-miR-205, ssc-miR-374a-3p and ssc-miR-451) and analyzed by qPCR to confirm their expression levels. To avoid known biases derived from sample hemolysis, free hemoglobin (absorbance at 414 nm) was measured in all samples with a NanoDrop 2000 Spectrophotometer (Thermo Fisher Scientific, Vienna, Austria) prior to qPCR analysis. An absorbance of A414 > 0.2 was considered indicative of hemolysis [58] and samples were excluded from qPCR analysis. Serum samples from the three treatment groups collected on d0 (Control *n* = 9, DON_LOW *n* = 8, DON_HIGH *n* = 10), d14 (*n* = 10/group), d26 (*n* = 10/group except for DON_LOW with *n* = 7) and d35/36 (*n* = 5/group) were analyzed. RNA was extracted as described for the serum samples of d26. cDNA was synthesized using the miRCury LNA RT Kit (Qiagen, Germany, Table S7). In total, 2 μL of purified RNA were used per 10 μL RT reaction, to which a defined molar amount of the non-mammalian microRNA cel-miR-39 was added to be used cDNA Spike-In. Real-time PCR amplification was performed in a 96-well plate format in a Roche LC480 II instrument (Roche, Germany) and miRCURY SYBR^®^ Green mastermix (Qiagen, Germany) with the following settings: 95 °C for 10 min, 45 cycles of 95 °C for 10 s and 60 °C for 60 s, followed by melting curve analysis. For one animal in the DON_HIGH group, two samples failed the qPCR quality control and, therefore, these and the corresponding samples at other time points were removed from further analysis.

### 4.4. Bioinformatics and Data Analysis

#### 4.4.1. MicroRNA Sequencing Data

Sequencing data were processed via an in-house developed pipeline. Raw reads were quality checked with the FastQC tool (v.0.11.4, www.bioinformatics.babraham.ac.uk/projects/fastqc/, last access date: 24 September 2021). Adaptors were trimmed using BBDuk from the BBMap toolkit v38.22 (minlen = 18 maxlen = 25 qtrim = rl trimq = 20 ktrim = r k = 21 mink = 11 hdist = 2), keeping only sequences between 18–25 bp long (the expected size range of microRNAs). For microRNA identification, the trimmed and quality filtered reads were aligned with bowtie (v1.2.2, https://github.com/BenLangmead/bowtie/releases/tag/v1.2.2_p1, last access date: 5 November 2021) [59] to the miRbase v21 (http://www.mirbase.org/, last access date: 20 May 2021) [60], specifically to the microRNAs of *Sus scrofa* (genome assembly 10.2). A count table of reads mapping to each microRNA was used for downstream analyses.

Log-transformed counts of sequencing reads per microRNA per sample were used for principal component analysis (PCA) to evaluate the clustering of samples based on their microRNA expression profiles. Raw counts were used for differential expression analysis via DESeq2 package in R (v3.5.2, https://www.r-project.org/, last access date: 5 November 2021) [61] to identify microRNA with significant differences in expression between the treatment groups. The *p*-values obtained were adjusted for multiple testing using Benjamini–Hochberg false discovery rate (FDR). Adjusted *p*-values < 0.1 (FDR 10%) were considered significant. All analyses and plots were performed in R.

#### 4.4.2. MicroRNA qPCR Data

The microRNA expression from qPCR was estimated based on the cycle of quantification values (Ct-values). Ct-values were normalized to the UniSp4 exogenous spike-in. To remove any baseline differences in the microRNA abundances over time, the Ct-values were further normalized against the d0 values. To this end, for each sample, the individual microRNA Ct-values of d14, d28, and d35/36 were subtracted from the Ct-value of d0. Statistical analysis was performed by comparing the Ct-values between the treatment groups and the Controls using a two-way ANOVA followed by Tukey’s multiple comparison tests.

Receiver operating characteristic (ROC) curves were generated for the serum sequencing data using ratios of the upregulated versus the downregulated microRNA abundances based on read counts. Additionally, for the qPCR data, ROC curves were produced using the normalized Ct-values for each individual microRNA, and the area under the curve (AUC) was calculated to assess the accuracy of the models. To assess the predictive value of the combination of the four significant microRNAs, a multivariable logistic regression analysis was used, and the AUC for the predicted probability of the combined microRNAs signal was determined. Samples from the DON_LOW and the DON_HIGH were considered the treated samples and compared to the Controls. The regression was performed with the R glm package. ROC analyses were performed using the R pROC package [62].

#### 4.4.3. Target Gene Prediction

Target gene prediction for selected microRNAs was performed in DIANA miRPath (v.3.0, http://snf-515788.vm.okeanos.grnet.gr/, last access date: 5 November 2021) [63], specifically using the miRTarBase database [64] of experimentally validated targets for these microRNAs in humans (since there is no data available for pigs) and microT-CDS [65] for predictions based on sequence patterns (not experimentally validated). Enrichment for specific KEGG (Kyoto Encyclopedia of Genes and Genomes) pathways among the target genes identified by both prediction tools was performed using ShinyGO: Gene Ontology Enrichment Analysis (v0.66, http://bioinformatics.sdstate.edu/go/, last access date: 16 April 2021) [66] with a *p*-value cutoff (FDR) < 0.05. The relation between the enriched functional pathways was visualized through a network with nodes representing the pathways. Two pathways were shown connected if they shared at least 20% of their genes [66]. Gene Ontology annotations for biological functions of the target genes, and high-level GO Terms, were also generated with ShinyGO (v0.66, http://bioinformatics.sdstate.edu/go/, last access date: 16 April 2021).

## Figures and Tables

**Table 1:** Fold changes and adjusted *p*-values of microRNAs in serum samples significantly affected in the DON_LOW or DON_HIGH group compared to the Control. The eight microRNAs affected both in DON_LOW and DON_HIGH are indicated in bold. n.s., not significant (adjusted *p*-value > 0.1).

	MicroRNA ID	Fold-Change Treated vs. Control		Adj. *p*-Value vs. Control	
		DON_LOW	DON_HIGH	DON_LOW	DON_HIGH
Upregulated microRNAs	**ssc-miR-16**	**2.325**	**1.709**	**0.082**	**0.067**
	ssc-miR-17-5p	n.s.	2.288	n.s.	0.053
	ssc-miR-92a	n.s.	1.391	n.s.	0.067
	**ssc-miR-128**	**2.180**	**1.849**	**0.070**	**0.053**
	ssc-miR-148a-3p	n.s.	1.435	n.s.	0.014
	**ssc-miR-205**	**54.720**	**34.344**	**0.059**	**0.032**
	ssc-miR-339	2.540	n.s.	0.082	n.s.
	ssc-miR-339-5p	2.542	n.s.	0.082	n.s.
	ssc-miR-361-3p	4.064	n.s.	0.082	n.s.
	**ssc-miR-451**	**2.594**	**1.751**	**0.073**	**0.052**
Downregulated microRNAs	**ssc-miR-10b**	**0.457**	**0.593**	**0.059**	**0.033**
	ssc-miR-10a-5p	0.572	n.s.	0.082	n.s.
	ssc-miR-92b-3p	0.480	n.s.	0.082	n.s.
	**ssc-miR-99b**	**0.381**	**0.428**	**0.070**	**0.013**
	ssc-miR-103	n.s.	0.646	0.013	n.s.
	ssc-miR-107	n.s.	0.651	0.013	n.s.
	ssc-miR-126-5p	0.407	n.s.	0.059	n.s.
	**ssc-miR-192**	**0.456**	**0.526**	**0.082**	**0.017**
	**ssc-miR-374a-3p**	**0.414**	**0.368**	**0.070**	**0.016**
	ssc-miR-1388	0.412	n.s.	0.07	n.s.
	ssc-let-7a	n.s.	0.706	n.s.	0.097
	ssc-let-7f-5p	n.s.	0.706	n.s.	0.048
	ssc-let-7g	n.s.	1.582	n.s.	0.097

## Data Availability

The microRNA sequencing data generated during the current study are available in the Sequence Read Archive (SRA) repository, under BioProject PRJNA732548.

## Supplementary Materials

The following are available online at https://www.mdpi.com/article/10.3390/ijms222112043/s1.
