# Supplementary material for: MicroRNA Expression Profiling in Porcine Liver, Jejunum and Serum upon Dietary DON Exposure Reveals Candidate Toxicity Biomarkers
Source: Int J Mol Sci. 2021 Nov 7;22(21):12043. doi: 10.3390/ijms222112043 (PMC8585098; doi:10.3390/ijms222112043)
Supplement: Supplementary file 1 [file ijms-22-12043-s001.zip › ijms-1416748_SupplementaryFigures and Table S1.pdf]

Supplementary Materials

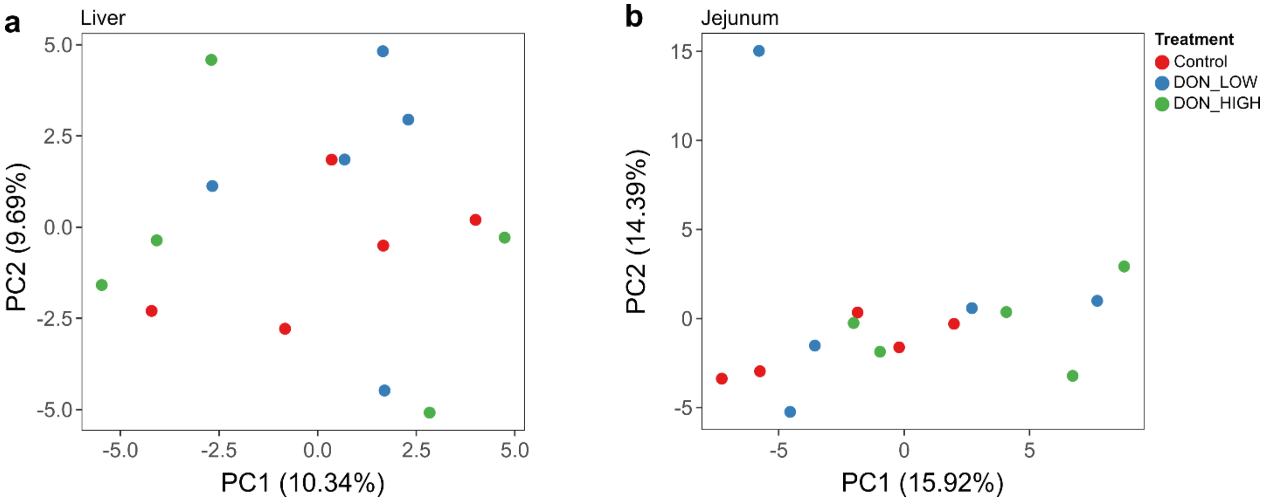

Figure S1. Principal component analysis based on microRNA abundance in (a) liver and (b) jejunum samples.

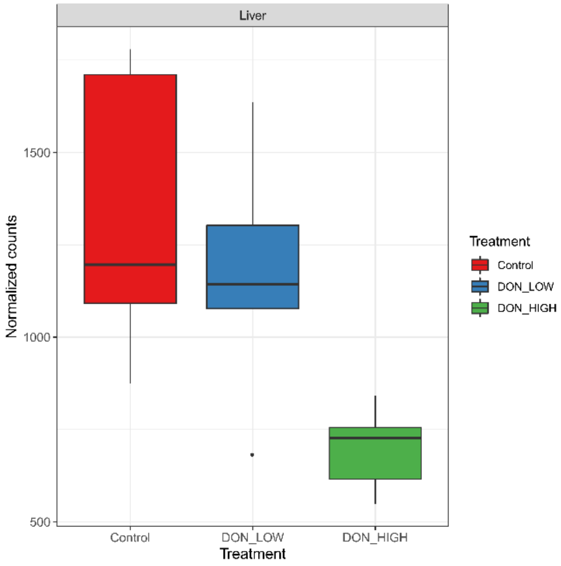

Figure S2. Normalized read counts for ssc-miR-10b in porcine liver samples from the Control and DON-exposed groups. The abundance of ssc-miR-10b was significantly lower in DON_HIGH treated samples (DESeq2 Log2FoldChange = -0.932 and FDR = 0.009).

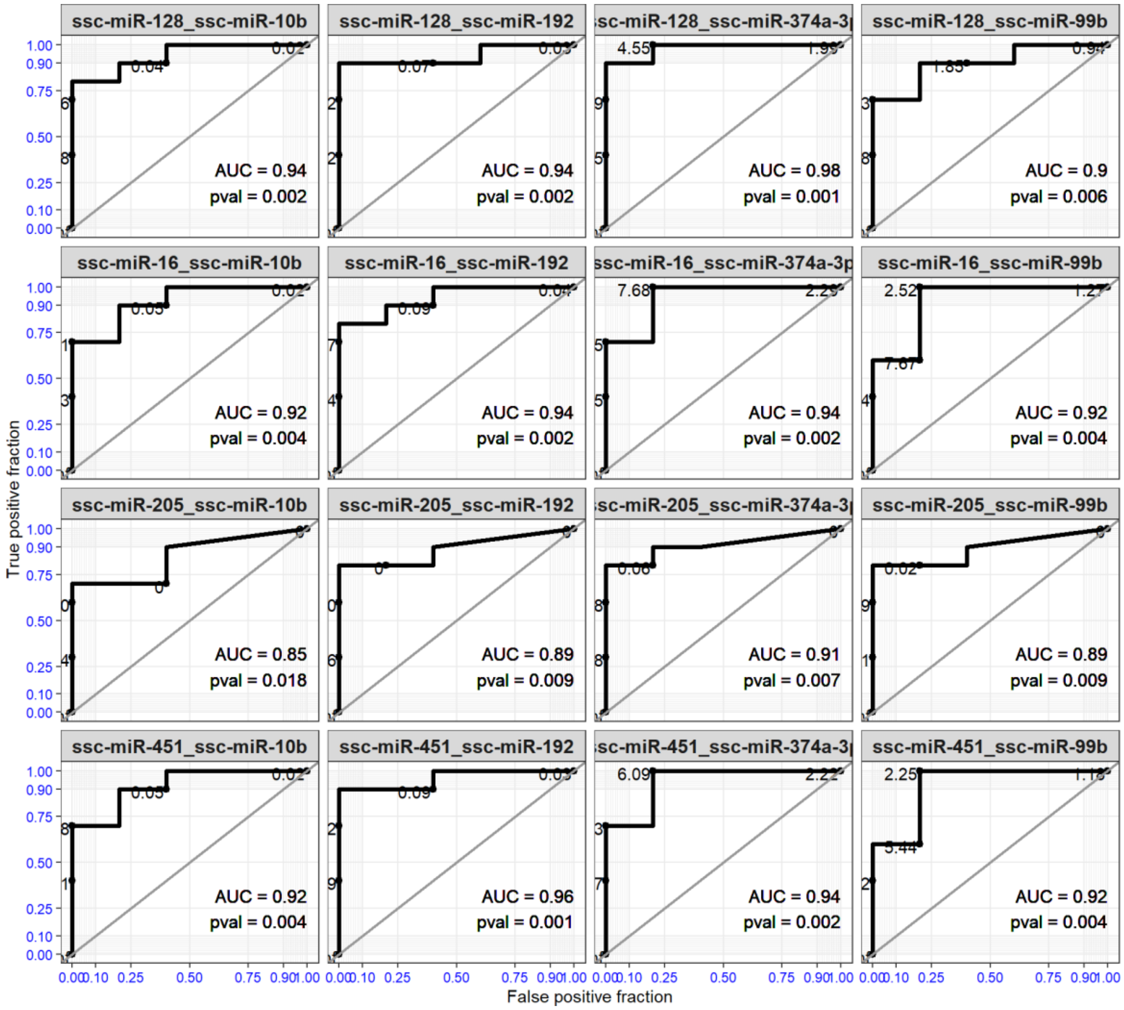

Figure S3. ROC curve analysis based on ratios derived from normalized counts of sequencing reads of differentially expressed microRNAs in the serum of DON-exposed pigs compared to the Controls.

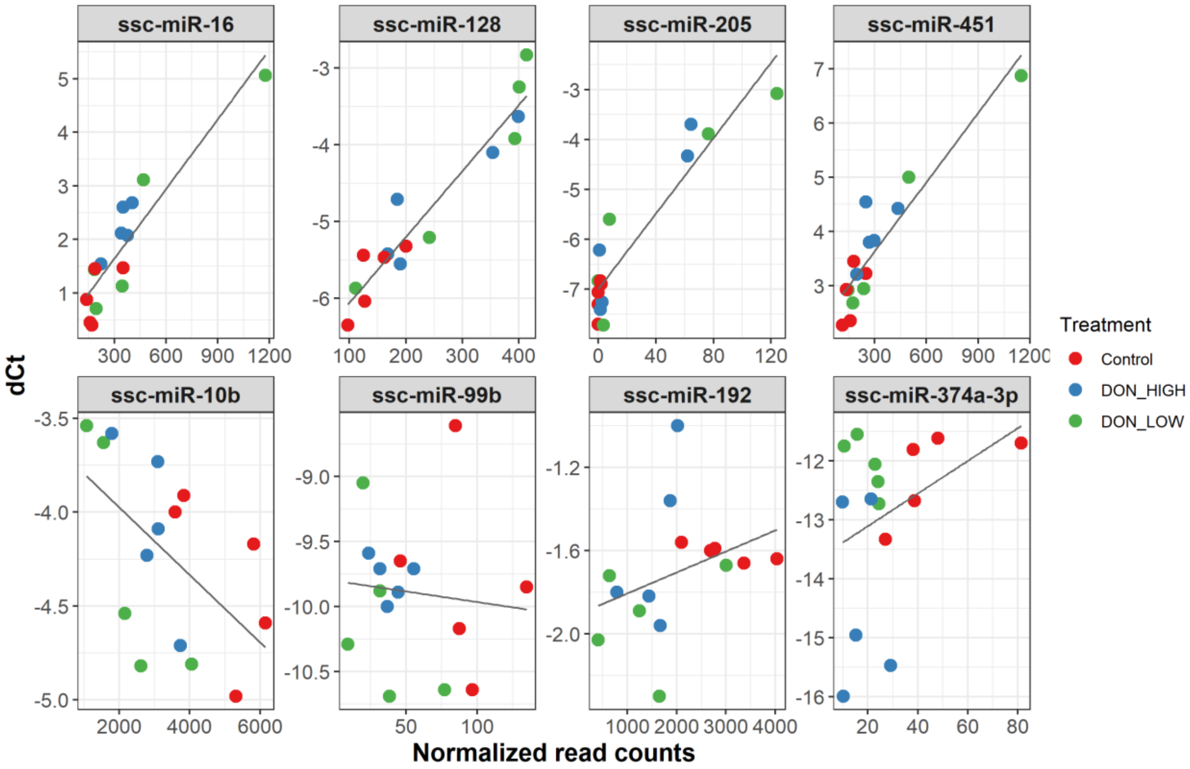

Figure S4. Correlation of microRNA abundance in the serum based on sequencing data and qPCR. Normalized read counts and normalized Ct-values are shown for each of the eight selected microRNAs. The four microRNAs found upregulated in the serum based on the sequencing data are shown in the top row, the downregulated microRNAs from the sequencing data are shown in the bottom row.

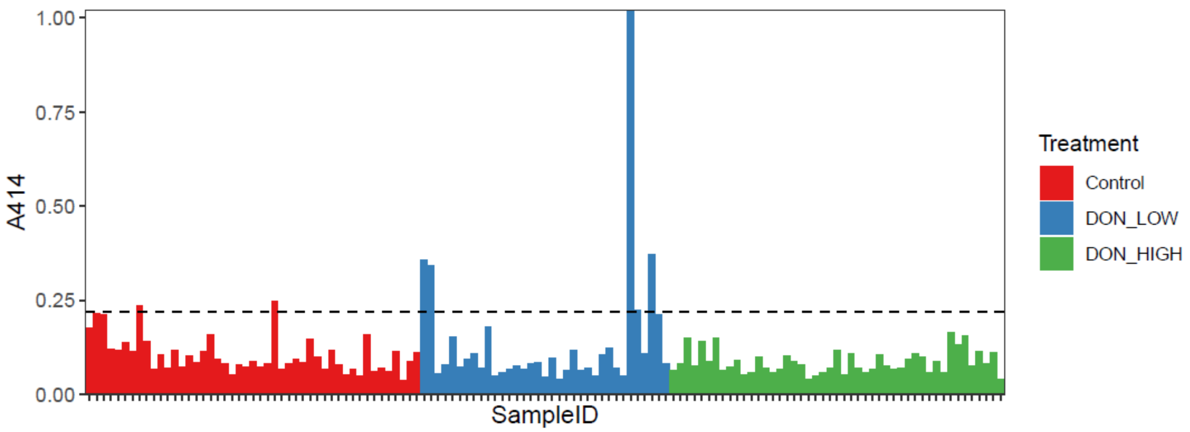

Figure S5. Absorbance of serum samples at A414 to identify hemolytic samples. The dotted line marks A414 = 0.2 used as a threshold based on Kirschner et al, PLoS One 2011.

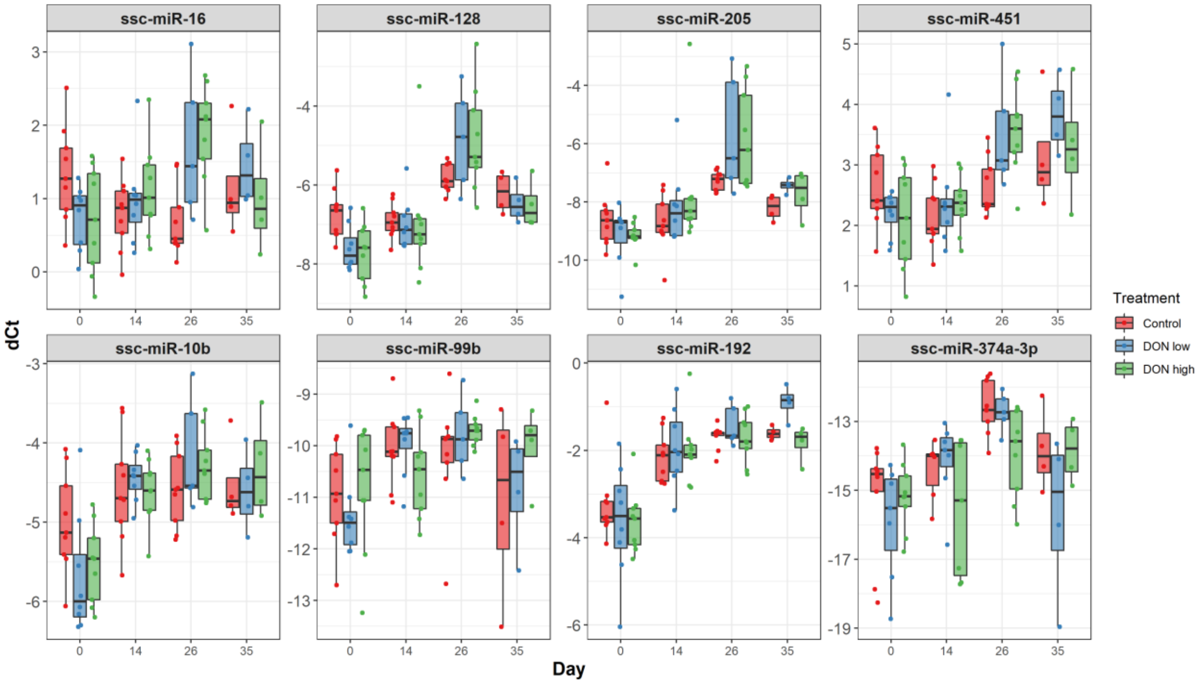

Figure S6. MicroRNA expression in the serum based on qPCR in different treatment groups. The raw dCt (normalized Ct-values) are shown without any correction for variations on d0.

a

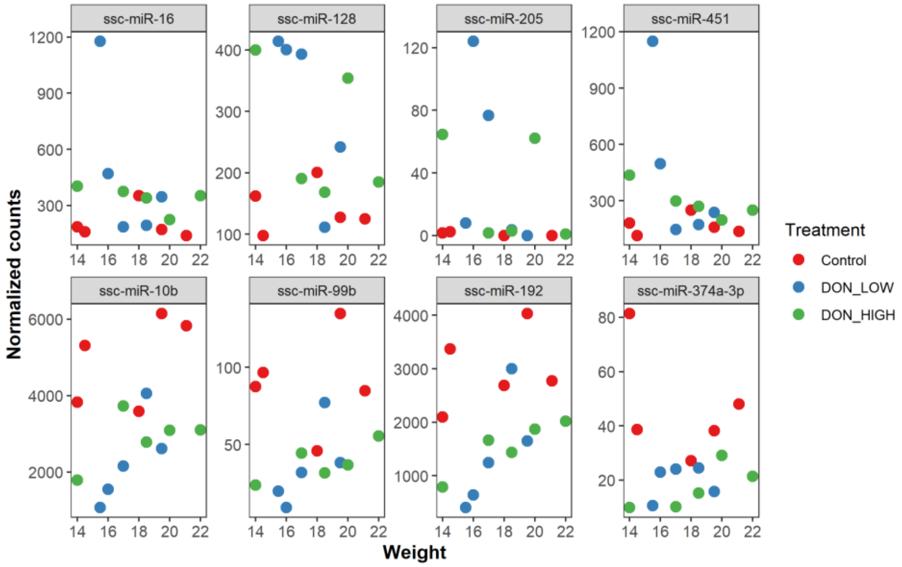

b

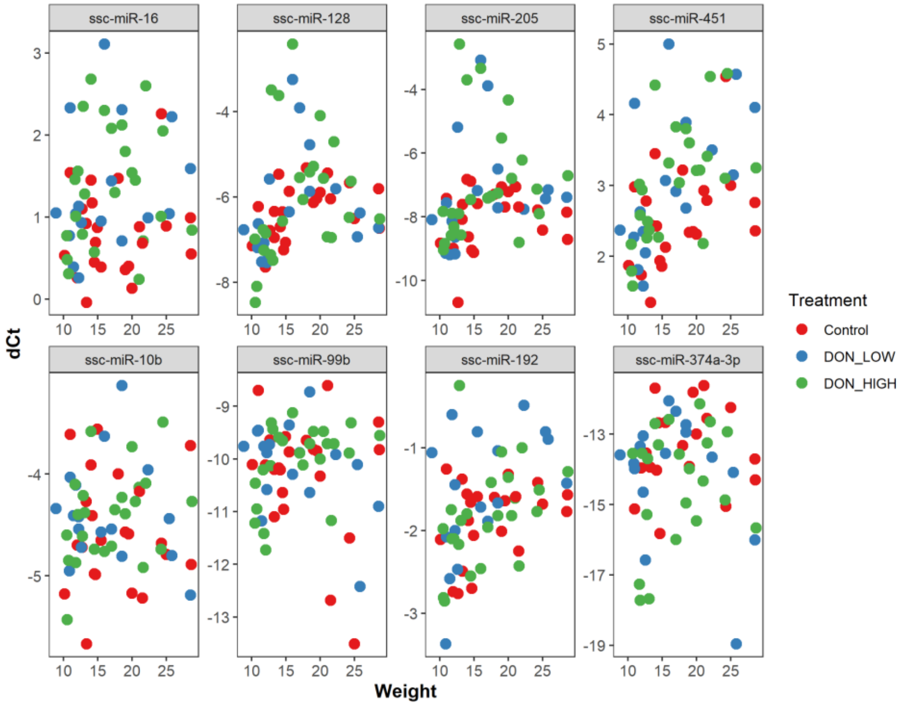

Figure S7. Relation between the weight of the animals and microRNA expression determined by (**a**) sequencing (samples at day 26), and (**b**) qPCR (including samples at all time points).

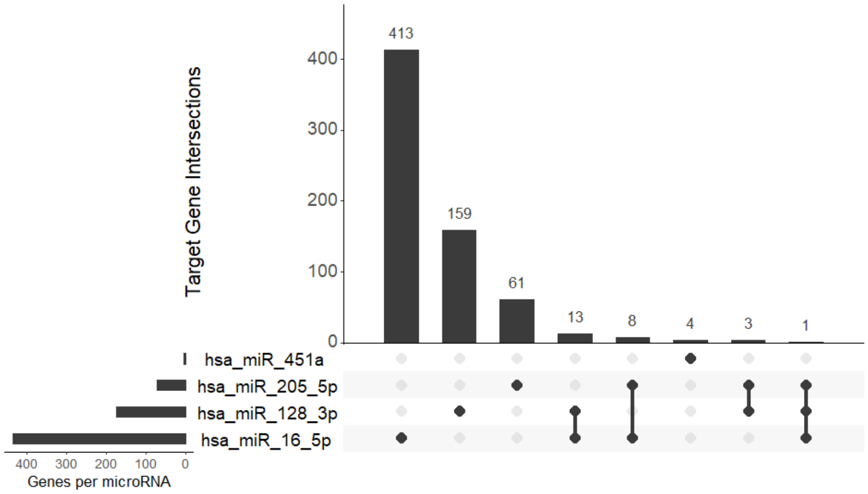

Figure S8. Total number of target genes for each microRNA and overlaps in the target gene sets between the different microRNAs. Horizontal bars show the total number of target genes for each microRNA and vertical bars indicate the number of target genes shared or unique to each microRNA.

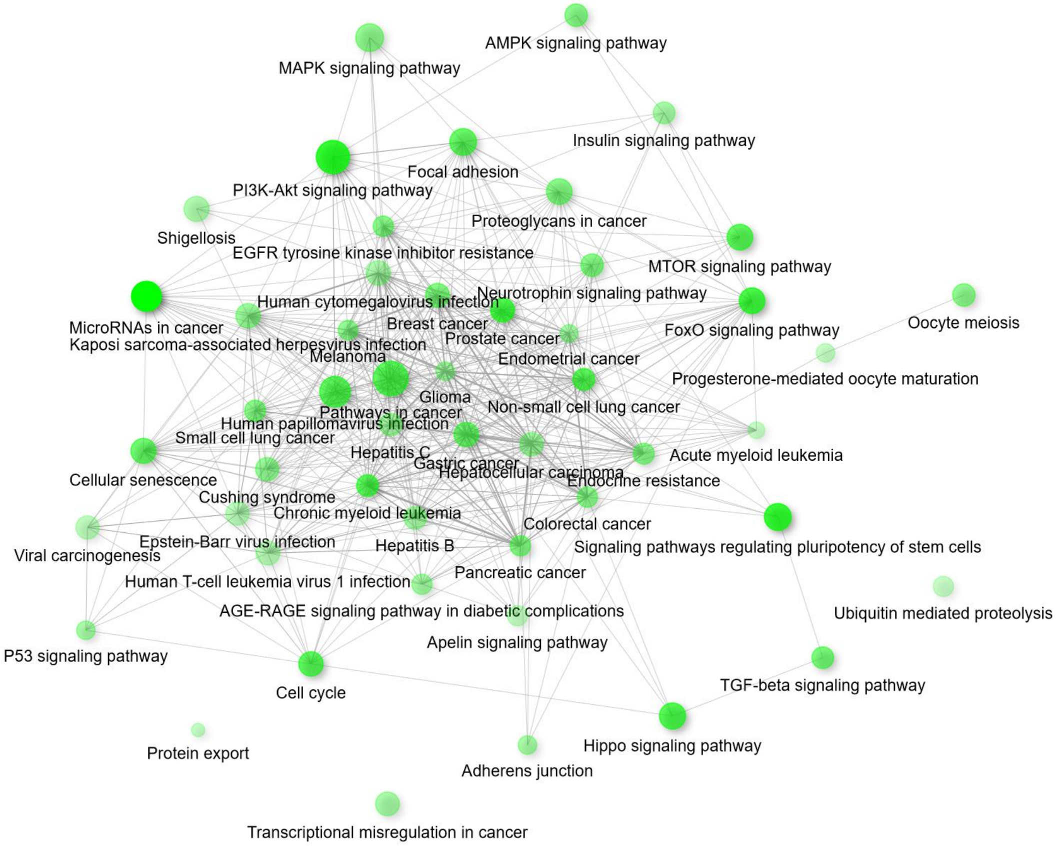

Figure S9. KEGG pathway enrichment network based on the target genes of the four upregulated microRNAs. The relation between top 50 enriched pathways are shown. Two pathways (nodes) are connected if they share at least 20% or more genes. Darker nodes are more significantly enriched gene sets. The size of the nodes represents the size of the gene sets and the edge thickness represents the number of overlapping genes.

Table S1. Studies investigating the effects of deoxynivalenol (DON) on the microRNA expression *in vitro* or *in vivo*.

Study		DON administration			MicroRNA analysis		Significant effects of DON treatment on microRNA expression compared to Control	Reference
**Type**	**Species**	**Route**	**Dose**	**Duration**	**Tissue**	**Approach**		
In vivo	Pig (female)	Oral (morning feed)	12 µg/kg body weight/day	7 - 42 days	Liver, duodenum, jejunum, ascending colon, descending colon	Targeted analysis: miR-9, miR-15a, miR-21, miR-34a, miR-122, miR-125b, miR-192	Downregulation of miR-15a in liver on day 21<br>Upregulation of miR-21 in ascending colon on day 7	Brzuzan et al., 2015
In vivo	Mouse (male)	Oral (gavage)	25 µg/kg body weight/day	90 days	Liver	Untargeted analysis	Upregulation of mmu-miR-7240-5p, mmu-miR-182-5p, mmu-miR-96-5p, mmu-miR-3470a, mmu-miR-708-3p, mmu-miR200a-5p, mmu-miR-224-5p	Liao et al., 2020 [2]
In vitro	Mouse (Hepa1-6)		0.2 - 1.4 µM	12 - 48 hours	Liver	Targeted analysis: mmu-miR-7240-5p	Upregulation of mmu-miR-7240-5p	
In vitro	Pig (IPEC-J2)		1.6 – 12.8 µg/mL	12 hours	Jejunum	Targeted analysis: miR-30c, miR-181-a, miR-365-5p, miR-769-3p	None observed	Xie et al., 2020 [3]
In vitro	Pig (IPEC-J2)		1.6 µg/mL	12 hours	Jejunum	Targeted analysis: miR-27a, miR-27b, miR-92a, miR-148a, miR-185, miR-191, miR-221, miR-222, miR-378	Downregulation of miR-92a, miR-221, miR-148a, miR-222<br>Upregulation of miR-185	Hou et al., 2021 [4]
In vivo*	Mouse (male)	Oral (gavage)	2.5 µg/kg body weight/day	4 weeks	Intestinal mucosa, intestinal submucosa, skeletal muscle, adipose tissue	Targeted analysis: miR-221, miR-222	Decrease of miR-221/miR-22 ratio in intestinal mucosa	

* Presence/absence of adenovirus-CDX2-promotor in DON group unclear based on description in experimental section

1. Brzuzan, P.; Woźny, M.; Wolińska-Nizioł, L.; Piasecka, A.; Florczyk, M.; Jakimiuk, E.; Góra, M.; Łuczyński, M.K.; Gajęcki, M. MicroRNA Expression Profiles in Liver and Colon of Sexually Immature Gilts after Exposure to Fusarium Mycotoxins. *Polish Journal of Veterinary Sciences* **2015**, *18*, 29–38, doi:10.1515/pjvs-2015-0004.
2. Liao, Y.; Peng, Z.; Wang, L.; Li, D.; Yue, J.; Liu, J.; Liang, C.; Liu, S.; Yan, H.; Nüssler, A.K.; et al. Long Noncoding RNA Gm20319, Acting as Competing Endogenous RNA, Regulated GNE Expression by Sponging MiR-7240-5p to Involve in Deoxynivalenol-Induced Liver Damage in Vitro. *Food and Chemical Toxicology* **2020**, *141*, 111435, doi:10.1016/j.fct.2020.111435.
3. Xie, M.; Chen, T.; Xi, Q.; Hou, L.; Luo, J.; Zeng, B.; Li, M.; Sun, J.; Zhang, Y. Porcine Milk Exosome MiRNAs Protect Intestinal Epithelial Cells against Deoxynivalenol-Induced Damage. *Biochemical Pharmacology* **2020**, *175*, 113898, doi:10.1016/j.bcp.2020.113898.
4. Hou, L.; Tong, X.; Lin, S.; Yu, M.; Ye, W.-C.; Xie, M. MiR-221/222 Ameliorates Deoxynivalenol-Induced Apoptosis and Proliferation Inhibition in Intestinal Epithelial Cells by Targeting PTEN. *Frontiers in Cell and Developmental Biology* **2021**, *9*, 1–16, doi:10.3389/fcell.2021.652939.
